# Flt-1-positive cells are cancer-stem like cells in colorectal carcinoma

**DOI:** 10.18632/oncotarget.19403

**Published:** 2017-07-19

**Authors:** Ye Huang, Yinpeng Huang, Di Liu, Tianyi Wang, Guang Bai

**Affiliations:** ^1^ Department of General Surgery, The First Affiliated Hospital of Jinzhou Medical University, Jinzhou 121000, China; ^2^ Department of Anesthesiology, The First Affiliated Hospital of Jinzhou Medical University, Jinzhou 121000, China; ^3^ Department of Oncology, The First Affiliated Hospital of Jinzhou Medical University, Jinzhou 121000, China

**Keywords:** cancer stem cells (CSCs), colorectal carcinoma (CRC), Flt-1

## Abstract

Recent evidence demonstrates an essential role of cancer stem cells (CSCs) in cancer initiation, progression, migration, metastasis as well as chemo-resistance. Nevertheless, identification of CSCs in different cancers has not been succeeded, since such CSCs are typically lack of a specific and unique marker. Therefore, the current strategy is basically using one or several markers to enrich CSCs, or to isolate CSC-like cells. Here, we showed that in clinically obtained colorectal carcinoma (CRC) specimens, Flt-1, the type 1 receptor for vascular endothelial growth factor A, was significantly upregulated. Moreover, more distal metastasis and poorer patient survival were detected in Flt-1^high^ CRC, compared to Flt1^low^ subjects. Two CRC cell lines were then labeled with both luciferase and red fluorescent protein (RFP) reporters. We found that in both lines, compared to Flt-1- CRC cells, Flt-1+ CRC cells generated significantly more tumor spheres in culture, appeared to be more resistant to fluorouracil-induced apoptosis, were more detectable in the circulation after subcutaneous transplantation, and had a higher chances to generate tumor after serial adoptive transplantation. Thus, we conclude that Flt-1 may be used as a surface marker to enrich CSC in CRC. Selective elimination of Flt-1+ CRC cells may improve the therapeutic outcome.

## INTRODUCTION

Colorectal carcinoma (CRC) is a common cancer in humans and its malignancy is largely attributable to the migration and metastasis of some cancer cells to form distal tumor [1]. Combined approach including surgical removal of primary tumor, endoscopic therapy, chemotherapy, and radiation has been shown to significantly improve the patient survival [2]. However, these therapies were found to be less effective on a subpopulation of CRC cells that process stem cell-like properties [3, 4]. Indeed, CRC has been recently shown to be sustained by specific cells called cancer stem cells (CSCs), which are supposed to be responsible for the majority of the cancer invasion, migration, metastasis and chemo-resistance [5].

Surface markers have been extensively studied for identification, isolation and elimination of CSCs in a panel of different cancers. Interestingly, same surface CSC markers were shared by a number of different cancers, but other surface CSC makers CSC appear to be only important for certain cancers [6]. In CRC, the most recognized CSC markers are prominin-1 (CD133) [7, 8], Lgr5 [9, 10], CD44 [11–13], and EphB2 [14–16]. However, ALDH1, CD24, CD26, CD44, CD90, CD133, CD166 and side population have also been used for enriching CSCs in CRC [17]. Nevertheless, the current purified “CSCs” in CRC are only enriched CSC population, and could only be regarded as CSC-like cells. Additional surface markers for CSCs in CRC are necessary for further purification of such a small and unique population in the total tumor mass.

Flt-1 is the type 1 receptor for vascular endothelial growth factor A (VEGF-A). Flt-1 has 2 ligands, VEGF-A and placental growth factor (PlGF) [18]. Binding of either VEGF-A or PlGF to Flt-1 induces activation of the receptor and subsequent signaling transduction cascades, leading to regulation of biological and pathological events associated with cell transformation, cell proliferation, cell apoptosis, cell migration, vascularization, inflammation and tissue remodeling [18]. All these events are critical for tumor initiation, progression, migration, metastasis as well as chemo-resistance. Flt-1 was known to be expressed in endothelial cells, monocyte/macrophages, and some cancer cells [19]. However, Flt-1 as a CSC marker has not been studied. Here, we studied Flt-1 as a CSC marker for enriching CSC cells in CRC.

## RESULTS

### CRC specimens with higher Flt-1 associate with poor patient survival

**Figure 1:**
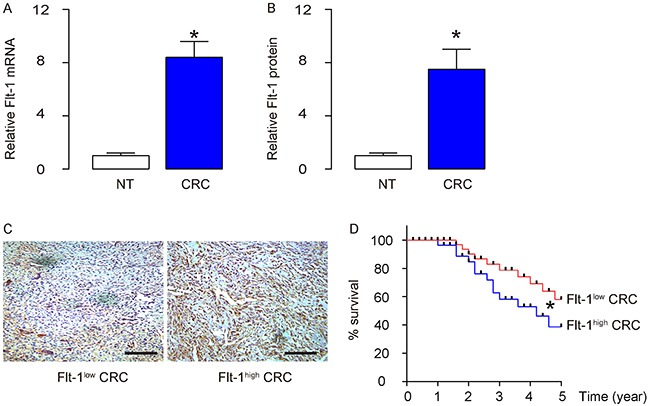
CRC specimens contain high Flt-1 associated poor patient survival **(A-B)** Flt-1 levels in 50 CRC (all at Stage III) specimens, and compared to the adjacent normal intestine tissue (NT), by RT-qPCR (A), and by ELISA (B). **(C)** Representative Flt-1 immunohistochemistry in Flt-1^high^ and Flt-1^low^ cases. **(D)** The 50 patients were followed-up for 5 years. The median value was chosen as the cutoff point for Flt-1^high^ cases (n=25) from Flt-1^low^ cases (n=25). Kaplan-Meier curves were generated. *p<0.05. N=50.

We examined the Flt-1 levels in 50 CRC (all at Stage III) specimens, and compared to the adjacent normal intestine tissue (NT). We detected higher levels of Flt-1 in CRC specimens, compared to NT, by RT-qPCR (Figure 1A), and by ELISA (Figure 1B). To figure out whether the levels of Flt-1 in the CRC specimens may correlate with overall survival of the patients, these 50 patients were followed-up for 5 years. The median value was chosen as the cutoff point for Flt-1^high^ cases (n=25) from Flt-1^low^ cases (n=25). Kaplan-Meier curves were generated, which showed that CRC patients with higher Flt-1 levels in the cancer had a significantly poorer overall survival (Figure 1C–1D).

### Expression of luciferase and RFP reporter in 2 CRC cell lines

**Figure 2:**
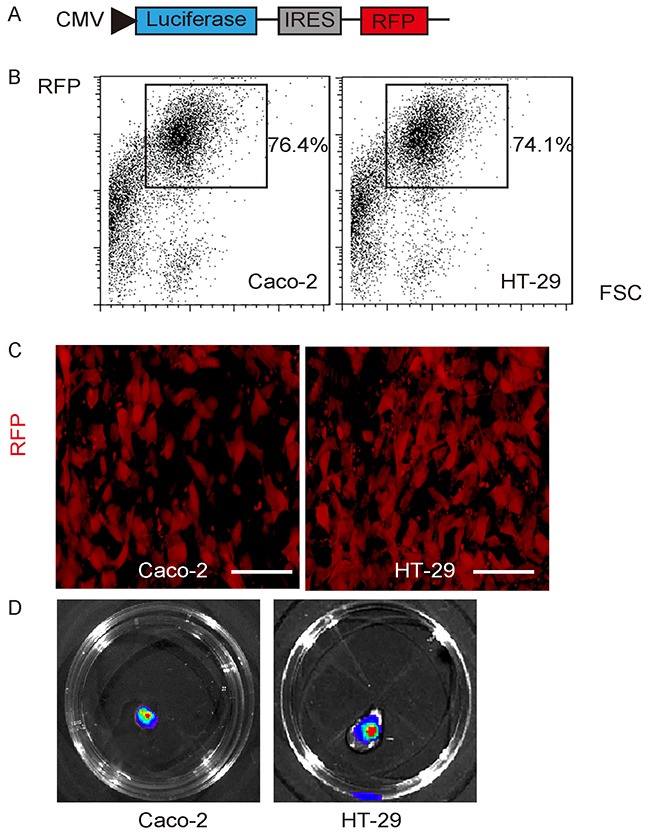
Expression of luciferase and RFP reporter in 2 CRC cell lines **(A)** Illustration of a lentivirus carrying both luciferase and red fluorescent protein (RFP) reporter under the control of a CMV promoter (Le-CMVp-LUC-RFP). **(B)** Two human CRC cell lines Caco-2 and HT-29 were transduced with Le-CMVp-LUC-RFP, after which the RFP+ cells from both lines were purified with flow cytometry, based on RFP. **(C)** The purified transduced cells were red fluorescent in culture. **(D)** The transduced cells were readily detectable after exposed to luciferin in culture. Scale bars are 20 μm.

Next, we aimed to examine the association of Flt-1 with CRC stemness. Two human CRC cell lines Caco-2 and HT-29 were transduced with a lentivirus carrying both luciferase and red fluorescent protein (RFP) reporter under the control of a CMV promoter, to allow *in vivo* tracing of cancer cells and tumor formation in living animals, and analysis and isolation of transplanted cancer cells from mice (Figure 2A). After lentiviral transduction, the RFP+ cells from both lines were purified with flow cytometry, based on RFP (Figure 2B). The purified transduced cells were red fluorescent in culture (Figure 2C), and were readily detectable after exposed to luciferin in culture (Figure 2D). The latter is the basis for *in vivo* tracing of the grafted cancer cells and tumor formation in living animals.

### Separation of Flt-1+ vs Flt-1- cells

**Figure 3:**
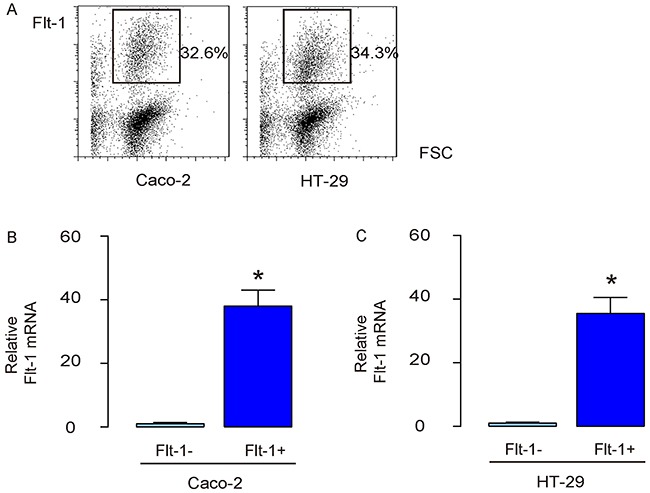
Separation of Flt-1+ vs Flt-1- cells **(A)** Flt-1 was used as a surface marker to separate Flt-1+ vs Flt-1- populations from the transduced CRC cells using flow cytometry. **(B-C)** RT-qPCR for Flt-1 in Flt-1+ vs Flt-1- populations from the transduced Caco-2 cells (B), and in transduced HT-29 cells (C). *p<0.05. N=5.

Flt-1 was used as a surface marker to separate Flt-1+ vs Flt-1- populations from the transduced CRC cells using flow cytometry (Figure 3A). After two fractions were obtained, the Flt-1 levels were examined by RT-qPCR, showing nearly 40 times higher Flt-1 levels in the Flt-1+ cells, compared to Flt-1- cells, in transduced Caco-2 cells (Figure 3B), and in transduced HT-29 cells (Figure 3C).

### Flt-1+ CRC cells demonstrate CSC properties *in vitro*

**Figure 4:**
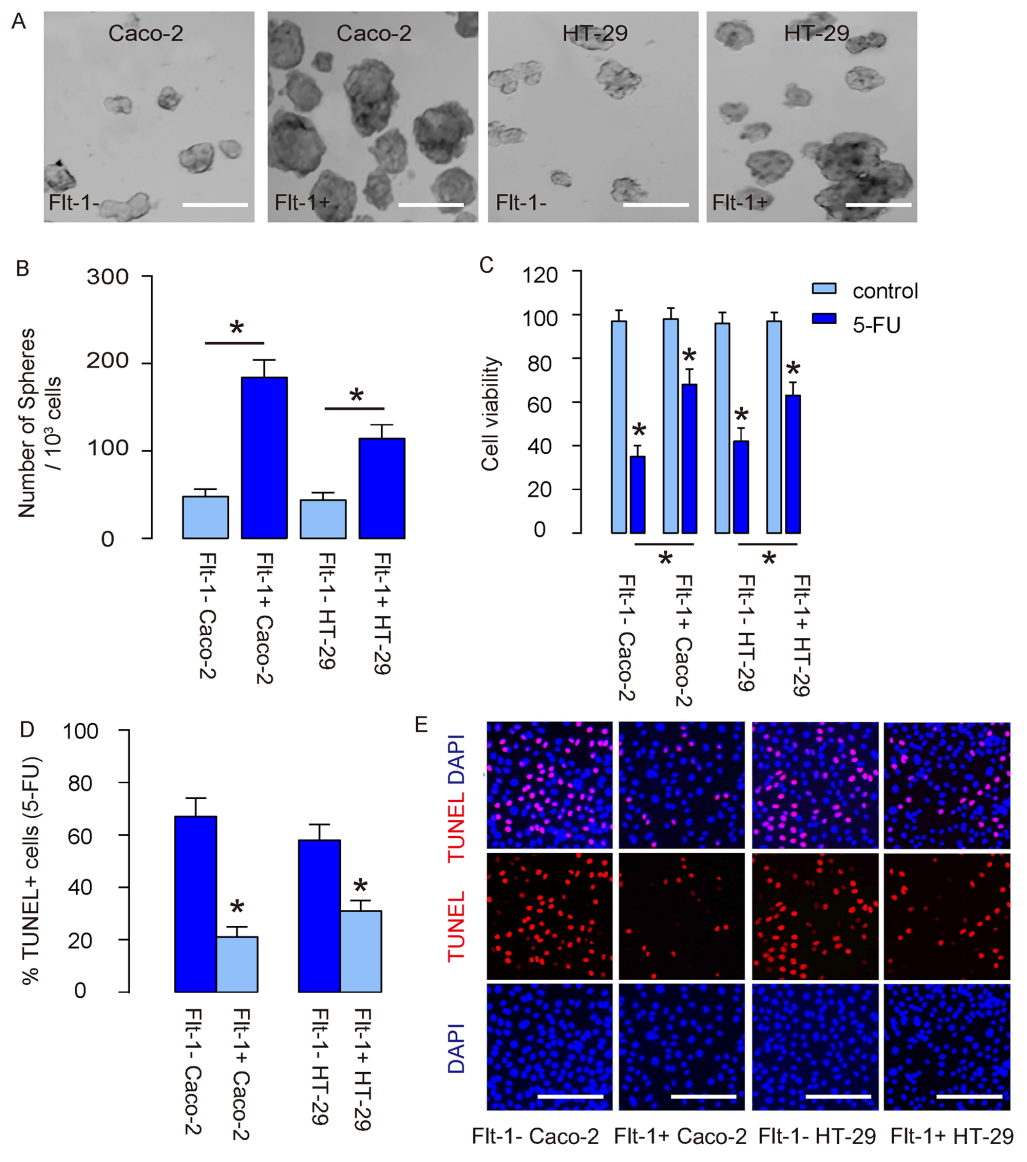
Flt-1+ CRC cells demonstrate CSC properties *in vitro* **(A)** Tumor sphere formation assay for Flt-1- and Flt-1+ cells from both lines, shown by representative images (A), and by quantification **(B)**. **(C-E)** Flt-1- and Flt-1+ cells from both lines were exposed to fluorouracil (5-FU). (C) CCK-8 assay for cell viability. (D-E) TUNEL assay for cell apoptosis, shown by quantification (D), and by representative staining images (E). *p<0.05. N=5. Scale bars are 100 μm.

Two strategies were then used to examine the CSC properties of Flt-1+ cells, using Flt-1- cells as controls. First, Flt-1- and Flt-1+ cells from both lines underwent in a tumor sphere formation assay. We found that compared to Flt-1- cells, Flt-1+ cells generated significantly more tumor spheres in both lines, shown by representative images (Figure 4A), and by quantification (Figure 4B). Next, Flt-1- and Flt-1+ cells from both lines were exposed to fluorouracil (5-FU), the first line chemotherapeutic drug for CRC, in an CCK-8 assay. We found that compared to Flt-1- cells, Flt-1+ cells had significantly better survival in the presence of 5-FU (Figure 4C), likely resulting from reduction in cell apoptosis examined by TUNEL assay, shown by quantification (Figure 4D), and by representative staining images (Figure 4E). Hence, Flt-1+ CRC cells demonstrate CSC properties *in vitro.*

### Transplanted Flt-1+ CRC cells generate bigger tumor vs Flt-1- CRC cells

**Figure 5:**
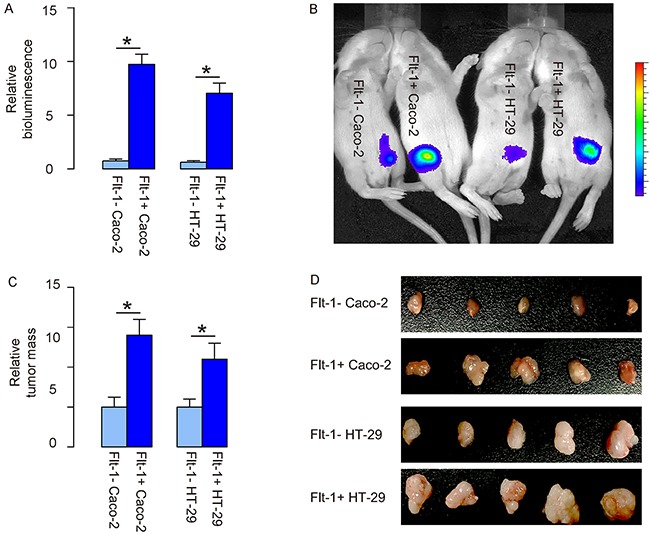
Transplanted Flt-1+ CRC cells generate bigger tumor vs Flt-1- CRC cells **(A-D)** Same number (10^6^) of Flt-1- and Flt-1+ cells was subcutaneously transplanted into nude mice, and the tumor formation was monitored after luciferin injection 8 weeks after tumor implantation. (A-B) Tumor size was analyzed by quantification of bioluminescence (A) and shown by the representative bioluminescent images (B). (C-D) Tumor mass after resection was measured, showing by quantification (C), and by gross images (D). *p<0.05. N=5.

Same number (10^6^) of Flt-1- and Flt-1+ cells was subcutaneously transplanted into nude mice, and the tumor formation was monitored after luciferin injection 8 weeks after tumor implantation. We found that compared to Flt-1- cells, Flt-1+ cells generated significantly larger tumor shown by quantification of bioluminescence (Figure 5A) and by the representative bioluminescent images (Figure 5B). Moreover, the tumor mass was detected after resection, showing that the tumor mass by Flt-1+ cells was significantly greater than those by Flt-1- cells (Figure 5C–5D).

### Flt-1+ CRC cells generate tumor more often than Flt-1- CRC cells after serial adoptive transplantation

**Figure 6:**
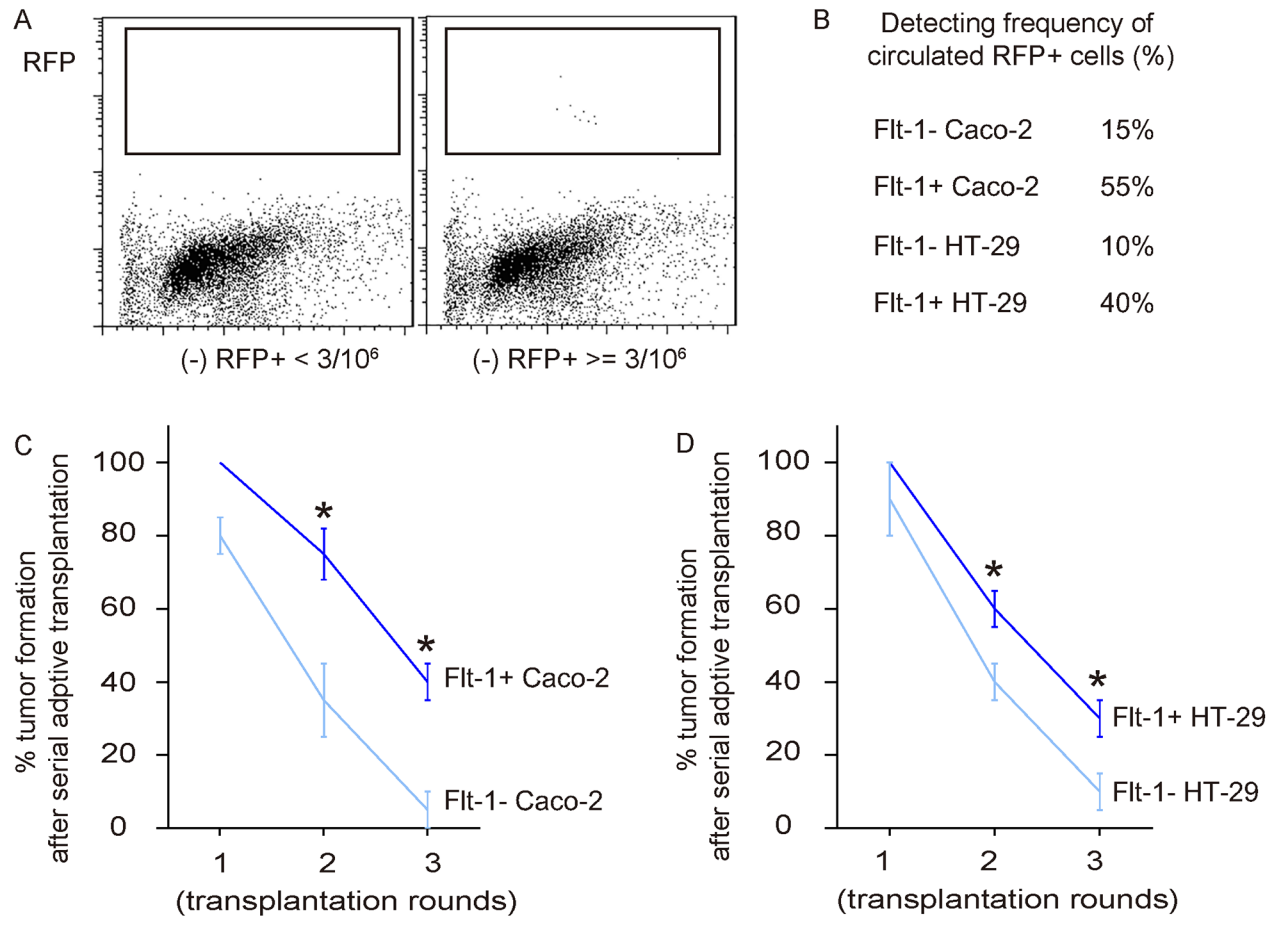
Flt-1+ CRC cells generate tumor more often than Flt-1- CRC cells after serial adoptive transplantation **(A)** Presence of RFP+ tumor cells in mouse blood 8 weeks after subcutaneous tumor cell transplantation by flow cytometry. Criteria was here: In 10^6^ blood cells that have deprived of red blood cells, if more than 3 RFP+ cells are detected, the case is regarded as a positive one. Otherwise, the case is regarded as a negative one. Representative flow charts for positive and negative cases were shown. **(B)** Frequency of detection of tumor cells in the circulation. **(C-D)** Finally, 20 tumor cells were isolated from the primary tumor developed from either Flt-1- and Flt-1+ cells, and were transplanted back to new nude mice. The new tumor formation was verified by bioluminescence. After 8 weeks, the newly formed tumors were dissected out and isolated 20 tumor cells were used for the second round of transplantation. Totally, 3 rounds of transplantation were performed and the formation of tumor was recorded throughout the experiment. Frequency of developing tumor by Caco-2 cells (C) and by HT-29 cells (D) were shown. *p<0.05. N=30.

In order to analyze the potential of detachment, migration and metastasis of Flt-1+ cells vs Flt-1- cells, we examined the presence of RFP+ tumor cells in mouse blood 8 weeks after subcutaneous tumor cell transplantation by flow cytometry. In 10^6^ blood cells that have deprived of red blood cells, if more than 3 RFP+ cells are detected, the case is regarded as a positive one. Otherwise, the case is regarded as a negative one (Figure 6A). We found that RFP+ tumor cells were more frequently detected in the circulation of mice transplanted with Flt-1+ CRC cells (Figure 6B). Finally, 20 tumor cells were isolated from the primary tumor developed from either Flt-1- and Flt-1+ cells, and were transplanted back to new nude mice. The new tumor formation was verified by bioluminescence. After 8 weeks, the newly formed tumors were dissected out and isolated 20 tumor cells were used for the second round of transplantation. Totally, 3 rounds of transplantation were performed and the formation of tumor was recorded throughout the experiment. We found that tumor was more often formed by Flt-1+ cells in the serial adoptive transplantation, compared to by Flt-1- cells (Figure 6C–6D). These *in vivo* data further support that Flt-1 purification enriches CSC cells in CRC.

## DISCUSSION

Development of a panel of chemotherapeutic and biological agents has improved the therapy for CRC, but most of these strategies are poor in removing and eliminating CSCs, compared to non-CSC cells, leading to treatment failure, chemo-resistance, and cancer recurrence [3, 4]. Consequently, CRC therapies targeting CSCs appear to be essential for further improvement of the outcome of the treatment and increases in the patients’ life span.

Different markers have been applied to characterize CSC in CRC. In 2007, CD133+ CRC cells were shown to have a CSC phenotype, and the mechanisms underlying CD133-mediated stemness include regulation of promoter methylation [7, 8]. Lgr5, a specific marker for intestinal stem cells, were later identified in CSCs of CRC [9], and were found to be associated with tumorigenicity [10]. Moreover, CD44 and CD166 were shown to be present on the surface of CSCs in CRC [11–13], while ALDH1, EpCAM and side population could also be used to enrich CSCs from CRC [14–17]. However, none of these markers are specific to CSCs. Thus, combination of different markers as well as searching for new markers on CSCs in CRC to increase the pool for identification may narrow down the selective candidate cells as CSCs.

In the current study, we found out that Flt-1 could be a novel surface marker for CSCs in CRC. Using both *in vitro* and *in vivo* gold standards for identification of CSCs, including tumor sphere formation, chemo-resistance and tumor formation in serial adoptive transplantation, we were able to show that the CSCs may be predominantly present in the Flt-1+ fraction. Since has 2 ligands, VEGF-A and PlGF [18], it is expected that the Flt-1 on CRC cells may interact with each other through VEGF/Flt-1 and/or PlGF/Flt-1-mediated signaling in a combined autocrine and paracrine way, to modulate cancer-associated vascularization and invasion. On the other hand, Flt-1 on CRC cells may also use these signaling pathways to crosstalk with tumor endothelial cells and inflammatory cells, to mediate not only vascularization-related biological and pathological events, but also cellular signal cascades to alter cell phenotype, control cell transformation, cell proliferation, cell apoptosis, cell migration and inflammation [18]. All these events are critical for maintenance of tumor cell stemness and CSC properties. These interactions among tumor cells and non-tumor cells inside the tumor highlight the importance of a microenvironment of cancer niche to the maintenance of cancer cell stemness. Moreover, Flt-1+ CRC cells were more frequently detected in the circulation, suggesting that Flt-1+ CRC cells may be not only enriched for CSCs, but also enriched for a fraction of circulating tumor stem-like cells [20].

We chose two commonly used human CRC lines in this study, which increased the reliability of the conclusion. Future studies may be applied to analyze the cell-cell interaction through Flt-1 signaling in CRC in a more detailed manner, which helps to understand the pathogenesis and tumorigenesis of CRC.

The relationship between clinicopathological factor and Flt-1 expression is still very controversial. For example, Flt-1 is overexpressed in primary tumors and nodal metastasis with no difference between primary and nodal metastasis [21]. On the other hand, loss of Flt-1 predicts distant metastasis (p = 0.026) and advanced stage (p = 0.049) of CRC [22], and is significantly associated with lymphogenous and hematogenous metastases [23]. Further studies are necessary for clarifying these questions.

To summarize here, our study demonstrates that Flt-1+ may be a novel CSC marker in CRC. Selective elimination of Flt-1+ CRC cells may improve the current CRC therapy.

## MATERIALS AND METHODS

### Protocol approval

All the experimental protocols including animal procedures have been approved by the research committee at the Jinzhou Medical University and carried out in accordance with the guideline. Resected CRC specimens were obtained together with the paired adjacent non-tumor intestine tissues (NT) from 50 patients since 2009 through 2012 at First Hospital of Jinzhou Medical University, with signed approval obtained from the involved patients.

### Cell culture and treatment

Two human CRC cell lines Caco-2 and HT-29 were both purchased from ATCC (American Type Culture Collection, Manassas, VA, USA). These cells were maintained in Dulbecco's Modified Eagle's Medium suppled with 20 % Fetal Bovine Serum (FBS, Sigma-Aldrich, San Jose, CA, USA) in a 37 °C incubator with 5 % CO_2_. 5-FU (Sigma-Aldrich) was dissolved in PBS to prepare a stock of 1mmol/l and applied to the culture at a final concentration of 2 μmol/l [24, 25]. Cells were analyzed 24 hours after treatment with 5-FU.

### Cell transduction and detection

The CRC cells were transduced with lentivirus carrying a red fluorescent protein (RFP) reporter and luciferase (LUC). A pcDNA3.1-CMV-RFP plasmid and a pcDNA3.1-CMV-luciferase plasmid were used as backbones (Clontech, Mountain View, CA, USA). Briefly, RFP coding construct was digested out with BamHI and Xhol, after which it was subcloned with an IRES, an internal ribosome entry site coding for RNA element that allows for translation initiation in an end-independent manner, into the pcDNA3.1-CMV-luciferase plasmid for generation of a pCMV-LUC-2A-RFP plasmid. For constructing lentiviral particles, HEK293T cells were co-transfected with pCMV-LUC-2A-RFP plasmid and 3 packaging plasmids (REV, pMDL and VSV-G) using Lipofectamine-3000 system (Invitrogen). The virus in supernatant was further processed, isolated and titrated. For *in vitro* transduction of CRC cells, a multiplicity of infection (MOI) of 100 was used and the incubation time was 48 hours to allow completeness of viral infection. Transduced cells were purified based on RFP expression by flow cytometry. The transduced cells were observed based on luciferase activity *in vitro,* after exposed to 150 μg/ml luciferin. The quantification of tumor mass in living animals used bioluminescence detection system (IVIS imaging system, Xenogen Corp., Alameda, CA, USA), 10 minutes after intraperitoneal injection of luciferin at 150 mg/kg body weight. The acquisition time was set to 1 minute and the binning value was 10.

### Animal manipulation

Male nude mice of 12 weeks of age (SLAC Laboratory Animal Co. Ltd, Shanghai, China) were used in the current study. Tumor cells were grafted subcutaneously and serial adoptive transfer was performed for 3 rounds with 20 cells isolated from the previous round. The bioluminescence was monitored 8 weeks after transplantation. The tumor formation was examined 8 weeks after transplantation using bioluminescence and measurement of dissected tumor.

### Primary tumor sphere culture

Sorted cancer cell fractions were re-suspended in tumor sphere media (TSM: DMEM suppled with 20 ng/ml human recombinant Epidermal growth factor, 20 ng/ml basic fibroblast growth factor, 10 ng/ml leukemia inhibitory factor and 60 μg/ml N-acetylcysteine). Afterwards, cells were seeded in ultra-low attachment 24-well plates (24 well plate coated with Ultra-Low Attachment Surface, Corning, NY, USA) at a density of 2×10^4^ cells per well. Formation of tumor sphere was examined 1 week after seeded.

### Cell viability assay

The cell viability was determined with CCK-8 detection kit (Sigma-Aldrich) at 450 nm with microplate reader, and calculated as the percentage of absorbance value in the examined well to the absorbance value in the control well.

### TUNEL Assay and immunohistochemistry

Terminal deoxynucleotidyl transferase (TdT)-mediated dUTP nick end labeling (TUNEL) was performed using a TUNEL Assay kit (R&D Biosystems, Shanghai, China). Nuclei were stained with DAPI (4′,6-diamidino-2-phenylindole, Sigma-Aldrich). Immunohistochemistry on tissue section was performed routinely using a rabbit-anti-human anti-Flt-1 antibody (R&D Biosystems) and the signals were detected by an ABC method (Dako, Shanghai, China).

### RT-qPCR

RT-qPCR was performed using QuantiTect SYBR Green PCR Kit (Qiagen, Shanghai, China), with the primers designed by Qiagen. A 2^-ΔΔCt^ method was used for quantification of gene expression levels. Relative expression levels of genes were obtained through sequential normalization of the values against β-actin and experimental controls.

### ELISA

ELISA for Flt-1 was performed using an ELISA kit (R&D Biosystems).

### Statistical analysis

The statistical analysis was performed with the GraphPad Prism 6 (GraphPad Software, San Diego, CA, USA). Comparison of 2 groups was carried out with Student's T test. All values represent the mean ± standard deviation (SD). A value of *p*<0.05 was considered as significant. Patients’ 5-year survival was recorded by Kaplan-Meier curve.
